# Traumatic Uterine Rupture in a Nonpregnant Patient: A Case Report

**DOI:** 10.1016/j.acepjo.2026.100457

**Published:** 2026-07-01

**Authors:** Akinori Takeda, Junpei Komagamine, Tomohiro Kurihara

**Affiliations:** Department of Emergency and Critical Care Medicine, National Hospital Organization (NHO) Tokyo Medical Center, Tokyo, Japan

**Keywords:** *pelvic fracture*, *traumatic uterine rupture*, *hemoperitoneum*, *cognitive error*, *diagnostic delay*

## Abstract

Traumatic uterine rupture is a rare and easily overlooked condition. Most cases of traumatic uterine rupture have been reported to occur in pregnancy. Here, we reported the case of a 14-year-old nonpregnant girl who presented to the emergency department with hemorrhagic shock due to multiple traumatic injuries after falling from a height during a suicide attempt. Initial resuscitation stabilized the patient's vital signs. A whole-body computed tomography (CT) scan revealed bilateral pneumothorax, multiple rib fractures, a grade III renal injury, a liver injury, and an unstable pelvic fracture. Although a poorly enhancing area was identified in the anterior wall of the uterine body on the initial CT scan, uterine rupture was not included in the active differential diagnosis at that time, as clinical priorities were directed toward other life-threatening injuries. Persistent unexplained hemoperitoneum led to a repeat CT scan performed on hospital day 4 and prompted magnetic resonance imaging on hospital day 7, which confirmed complete full-thickness rupture of the anterior uterine wall. Laparoscopic uterine repair was performed on hospital day 14. This case highlighted the importance of not dismissing uterine CT scan findings as incidental in female patients with unstable pelvic fractures, even when pregnancy has been excluded, and of considering pelvic magnetic resonance imaging scans when hemoperitoneum persists without a fully identified source. We also discussed the cognitive processes—including anchoring bias, confirmation bias, and task fixation (search satisficing)—that contributed to the delayed recognition of this diagnosis.

## 1 Introduction

Traumatic uterine rupture is a rare and easily overlooked condition that, if untreated, can compromise future fertility. It has been reported to occur in <0.5% of all cases of blunt abdominal trauma.^1^ The vast majority of reported cases occur in pregnancy,^2^, ^3^, ^4^ making traumatic uterine rupture in nonpregnant patients an exceedingly uncommon event. To our knowledge, only 3 prior cases of traumatic uterine rupture in nonpregnant women have been reported in the English-language literature.^5^, ^6^, ^7^ Here, we reported the fourth such case—and the first in a pediatric patient—involving a 14-year-old nonpregnant girl who presented with hemorrhagic shock due to multiple traumatic injuries, including uterine rupture, after falling from a height during a suicide attempt.

## 2 Case Presentation

A 14-year-old nonpregnant girl presented to our emergency department after falling from the rooftop of a 5-story apartment building (approximately 20 m above ground level) during a suicide attempt. During the fall, she collided with the knee of her mother, who attempted to catch her, sustaining direct blunt trauma to the lower abdomen. Her medical history included postencephalitic epilepsy, for which she had received a single course of pulse corticosteroid therapy at another hospital one year prior to this presentation; she was not on chronic corticosteroid therapy at the time of admission. Her current medications included levetiracetam, valproic acid, and lacosamide (a sodium channel-blocking adjunctive antiepileptic agent). She had no history of prior pregnancy and denied use of alcohol, cigarettes, or illicit drugs.

Upon arrival, the patient's airway was patent. Her breathing was labored, with a respiratory rate of 23 breaths per minute and an oxygen saturation of 83% on room air; supplemental oxygen at 10 L per minute via a nonrebreather mask was initiated. A chest radiograph revealed bilateral pneumothorax and multiple left rib fractures, and bilateral chest tube insertion stabilized her respiratory status. Her blood pressure was 102/77 mm Hg, and her heart rate was 122 beats per minute, with cold extremities consistent with hemorrhagic shock. A urine pregnancy test was negative. Vaginal bleeding was absent on examination. Initial laboratory evaluation revealed a hemoglobin level of 8.3 g/dL, a platelet count of 112,000/μL, and a lactate level of 5.1 mmol/L. Her initial Glasgow coma scale score was 12 (eye = 4; verbal = 3; and motor = 5); she was agitated and required sedation for pain and distress management, which limited the accuracy of abdominal examination. Her level of consciousness recovered after stabilization of her respiratory and hemodynamic status with bolus fluid infusion and blood transfusion.

Point-of-care ultrasound revealed free fluid in the abdomen and pelvis. Contrast-enhanced whole-body computed tomography (CT) scan revealed a left pulmonary contusion, bilateral pneumothorax, multiple rib fractures, a grade III left renal injury, a liver injury, and an unstable pelvic fracture with a sacral fracture and bilateral fractures of the pubis and ischium (Fig 1). There was no active contrast extravasation identified from any of these injuries, likely reflecting the effect of prior resuscitation. Although a poorly enhancing area was observed in the anterior wall of the uterine body on CT scan (Fig 1C), this finding was noted in the radiologic review but was not incorporated into the active differential diagnosis at that time, as the patient had been hemodynamically stabilized and clinical priorities were directed toward management of the unstable pelvic fracture and other life-threatening injuries. The patient was admitted to the intensive care unit.

**Figure 1:**
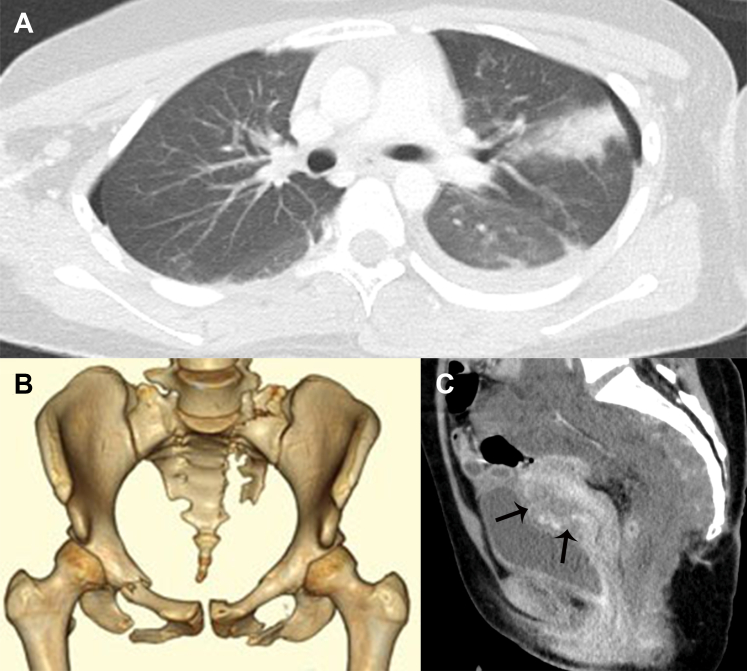
A, Computed tomography (CT) scan of the chest revealing left pulmonary contusion, left hemothorax, and bilateral pneumothorax, illustrating the severity of the thoracic injuries that competed for clinical priority during initial resuscitation. B, Three-dimensional CT scan reconstruction of the pelvis showing sacral fracture and bilateral fractures of the pubis and ischium, consistent with an unstable pelvic ring injury. C, Contrast-enhanced sagittal CT scan of the pelvis revealing a poorly enhancing area in the anterior wall of the uterine body (arrows). The bladder is visible anterior to the uterus as a low-density structure.

Throughout her intensive care unit stay, her hemodynamic status remained stable without evidence of recurrent shock. Definitive operative fixation of the pelvic fracture was performed on hospital day 6, and bilateral chest tubes were removed on hospital day 9 following resolution of traumatic pneumothorax. A repeat contrast-enhanced abdominal CT scan performed on hospital day 4, prompted by the absence of a clear explanation for the initial hemoperitoneum, revealed persistent fluid collection in the pelvic cavity despite overall hemodynamic stability. This finding prompted further evaluation with a magnetic resonance imaging (MRI) scan of the pelvis on hospital day 7, which revealed complete full-thickness rupture of the anterior uterine wall involving the endometrium, myometrium, and visceral peritoneum (Fig 2). Laparoscopic uterine repair was subsequently performed on hospital day 14. Intraoperative findings confirmed a full-thickness tear of the anterior uterine body. Her postoperative course was uneventful, and she was transferred to a rehabilitation facility on hospital day 49.

**Figure 2:**
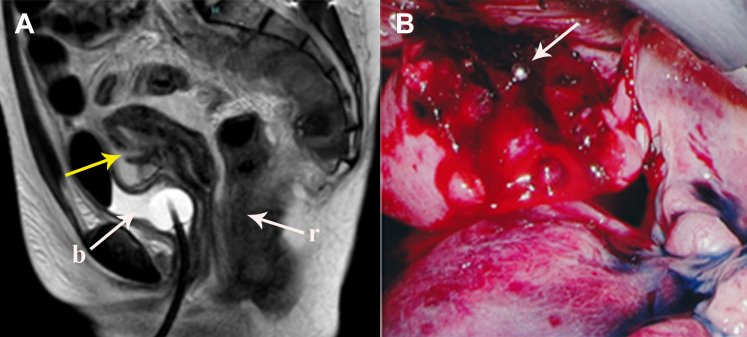
A, Sagittal T2-weighted magnetic resonance imaging scan of the pelvis demonstrating full-thickness rupture of the anterior uterine wall (yellow arrow), with clear disruption of the endometrium, myometrium, and visceral peritoneum. The bladder (b) and rectum (r) are labeled for anatomical orientation. B, Intraoperative photograph confirming complete rupture of the anterior wall of the uterine body. A uterine sound inserted transvaginally is visible through the rupture site (white arrow).

## 3 Discussion

Traumatic uterine rupture among nonpregnant women is exceedingly rare. To our knowledge, the present case represents the fourth reported case of traumatic uterine rupture in nonpregnant women and the first reported pediatric case. Three prior cases have been described: Kalstone and Laros^5^ reported a 39-year-old woman who sustained uterine rupture, bladder injury, and unstable pelvic fracture in a traffic accident; Grossberg and Druitt^6^ described a 23-year-old woman who experienced uterine rupture and bladder injury after becoming lodged between a boat deck and a jetty; and Zhu and Jin^7^ reported a 22-year-old woman with uterine rupture and unstable pelvic fracture following a road traffic accident. Because 3 of the 4 reported cases—including ours—were associated with unstable pelvic fractures, we hypothesize that a strong external force transmitted through the disrupted pelvic ring may be sufficient to cause uterine rupture even in the nonpregnant state.

Pathophysiologically, the nonpregnant uterus is relatively protected from blunt trauma by its small size, thick muscular wall, and surrounding pelvic bony architecture. However, when high-energy forces disrupt the pelvic ring—as occurs with unstable pelvic fractures—direct compressive or shearing forces may be transmitted to the uterus. The anterior uterine wall, which is less supported by surrounding structures than the posterior wall, may be particularly vulnerable. Corticosteroids have been reported to impair collagen synthesis and reduce tissue tensile strength, potentially increasing susceptibility to uterine wall injury.^8^^,^^9^ Although our patient's corticosteroid exposure was limited to a single pulse course 1 year prior to admission, we cannot exclude the possibility that residual effects on uterine wall integrity contributed to rupture in the setting of high-energy trauma.

A critical and instructive feature of this case is the cognitive and diagnostic process surrounding the delayed identification of uterine rupture. Importantly, the abnormal CT scan finding—a poorly enhancing area in the anterior uterine wall—was recognized on initial imaging. This was therefore not a case of a missed radiologic finding, but rather a case in which a recognized abnormality was not integrated into the clinical decision-making framework during the initial trauma resuscitation. This distinction is clinically important and warrants analysis through the lens of medical decision-making and cognitive error theory.^10^

At the time of initial evaluation, the emergency team appropriately anchored on the most immediately life-threatening diagnoses: hemorrhagic shock, bilateral pneumothorax, unstable pelvic fracture, renal injury, and liver injury. This anchor diagnosis—high-energy polytrauma without active uterine rupture—was reasonable given the overall clinical picture and the absence of vaginal bleeding, active contrast extravasation from the uterus, and hemodynamic deterioration attributable specifically to a uterine source. In the framework described by Croskerry,^10^ the initial anchoring was justifiable. However, the failure to subsequently adjust from this anchor—despite the unexplained uterine CT scan finding—represents a form of premature closure: once the patient was stabilized and transferred to the intensive care unit, the abnormal uterine finding was not re-evaluated as a potential source of the ongoing hemoperitoneum.

Several cognitive biases may have contributed to this delayed adjustment. First, anchoring bias led the clinical team to remain fixed on the initial framework of “no uterine rupture”—a reasonable assumption in a nonpregnant adolescent, given the exceptional rarity of the injury in this population. Second, confirmation bias may have reinforced this framework: the absence of vaginal bleeding and sustained hemodynamic stability were interpreted as evidence against significant uterine injury, whereas the CT scan finding was implicitly treated as incidental. Third, task fixation (also known as search satisficing)—the cognitive narrowing that occurs when clinicians concentrate on a specific high-priority problem such as pelvic fracture management—may have limited bandwidth for re-evaluating unexplained findings. These biases, operating together, delayed the diagnostic adjustment that the persistent hemoperitoneum ultimately demanded.

The diagnosis was ultimately reached through an appropriate, if delayed, process of systematic re-evaluation: the persistence of unexplained pelvic hemoperitoneum on repeat CT scan (hospital day 4) in a hemodynamically stable patient whose other injuries had been accounted for appropriately prompted an MRI scan, which confirmed the diagnosis. This reflects the principle that unexplained findings—particularly persistent fluid collections beyond the expected timeline of known injuries—should serve as a trigger for reassessment. The practical implication is not that a pelvic MRI scan should be performed routinely in all female patients with pelvic fractures, but rather that a low threshold for additional evaluation is warranted when (1) hemoperitoneum persists or is disproportionate to known injuries; (2) an unexplained uterine abnormality is present on the initial CT scan; or (3) hemodynamic instability cannot be fully attributed to identified sources. In such cases, a pelvic MRI scan is the most sensitive modality for confirming or excluding uterine rupture.

Cases of even longer diagnostic delay have been previously reported, with 2 cases diagnosed 2 to 6 years after the initial traumatic episode^11^^,^^12^—both of which involved pelvic trauma—underscoring the capacity for this injury to be entirely overlooked in nonpregnant women.

In conclusion, we reported the fourth documented case of traumatic uterine rupture in a nonpregnant woman and the first in a pediatric patient. This case illustrates that apparently incidental uterine CT scan findings should not be dismissed in women who have sustained high-energy pelvic trauma with an unstable pelvic fracture, even when pregnancy has been excluded, and hemodynamic stability has been achieved. When persistent unexplained hemoperitoneum is present, pelvic MRI scan should be considered as the next diagnostic step to avoid a preventable delay in diagnosis.

## Funding and Support

By *JACEP Open* policy, all authors are required to disclose any and all commercial, financial, and other relationships in any way related to the subject of this article as per ICMJE conflict of interest guidelines (see www.icmje.org). The authors have stated that no such relationships exist.

## Conflict of Interest

All authors have affirmed they have no conflicts of interest to declare.
